# Performance Differences of Hexavalent Chromium Adsorbents Caused by Graphene Oxide Drying Process

**DOI:** 10.1038/s41598-020-61760-2

**Published:** 2020-03-17

**Authors:** JinHyeong Lee, Hee-Gon Kim, Jung-Hyun Lee, So-Hye Cho, Kyung-Won Jung, Seung Yong Lee, Jae-Woo Choi

**Affiliations:** 1 Materials Architecturing Research Center, Korea Institute of Science and Technology, Hwarang-ro 14-gil 5, Seongbuk-gu Seoul, 02792 Republic of Korea; 2 Department of Chemical and Biological Engineering, Korea University, 145 Anam-ro, Seongbuk-gu Seoul, 02841 Republic of Korea; 3 Water Cycle Research Center, Korea Institute of Science and Technology, Hwarang-ro 14-gil 5, Seongbuk-gu Seoul, 02792 Republic of Korea; 4 Division of Nano & Information Technology, KIST school, Korea University of Science and Technology, Hwarang-ro 14-gil 5, Seongbuk-gu Seoul, 02792 Republic of Korea; 5 Division of Energy & Environment Technology, KIST school, Korea University of Science and Technology, Hwarang-ro 14-gil 5, Seongbuk-gu Seoul, 02792 Republic of Korea

**Keywords:** Engineering, Materials science

## Abstract

In this study, the influence of drying conditions on amine (−NH_3_) functionalization of graphene oxide (GO) was evaluated, and the hexavalent chromium (Cr(VI)) adsorption efficiency of the prepared materials was compared. 3-[2-(2-aminoehtylamino) ethylamino]propyl-trimethoxysilane (3N) was used for amine functionalization. The synthesized materials were analyzed by SEM, BET, TGA, XPS, and EA. TGA results showed that the solution-GO (SGO) was functionalized by more 3N molecules than freeze-dried GO (FDGO) and oven-dried GO (ODGO). Additionally, XPS analysis also showed that the ratio of N/C and Si/C was relatively high in SGO than FDGO and ODGO. The maximum adsorption capacity of SGO, FDGO, and ODGO for Cr(VI) was 258.48, 212.46, and 173.45 mg g^−1^, respectively. These results indicate that it is better to use SGO without drying processes for efficient amine functionalization and Cr(VI) removal. However, when the drying process is required, freeze-drying is better than oven-drying.

## Introduction

The active development in various industrial sectors, such as textile, metal finishing, leather tanning, stainless steel, and electroplating^1–7^ contribute to the discharge of contaminated wastewater containing inorganic (heavy metals, phosphates, nitrates, sulfates, etc.), organic (phenols, dyes, pesticides, pharmaceutical compounds, etc.), and biological (bacteria, viruses, etc.) compounds. Among these, heavy metals such as chromium (Cr), arsenic (As), copper (Cu), lead (Pb), cadmium (Cd), and mercury (Hg) are typical toxic substances that exist as cations and anions in water^8^. Chromium exists in two stable oxidation states, which are the hexavalent chromium (Cr(VI)) and trivalent chromium (Cr(III)) variants^9^. While Cr(III) is minimally toxic, Cr(VI) is highly toxic and causes significant environmental damage^10^. Further, Cr(VI) can cause carcinogenesis and mutations in humans. For these reasons, the World Health Organization (WHO) regulates the concentration of Cr(VI) in drinking water to less than 50 ng∙L^−1^ ^11^. To comply with this rigorous and necessary regulation, the application of water treatment techniques that can remove low concentrations of Cr(VI) is required. Various techniques, such as, redox, ion-exchange, adsorption, membrane filtration, and the like^12–16^ are applied according to the purpose. However, the removal of low concentrations of Cr(VI) with most of these wastewater treatment systems is highly challenging. Among these approaches, adsorption is more effective for Cr(VI) removal since adsorbents are eco-friendly and not generating by-products^17^. Therefore, we conducted further research on advancing adsorption technology for the removal of Cr(VI) from wastewater. For the first time, in this study, we developed an adsorbent based on graphene oxide (GO) and evaluated its Cr(VI)-adsorption performance.

GO has been used in various fields such as electronic, biological, and physical applications^18^ owing to its several advantages, such as straightforward synthesis, good solution stability, and the large surface-area-to-volume ratio^19–21^ and small weight-to-volume ratio. Particularly, the rich oxygen-containing surface functional groups (hydroxyl, epoxy, carbonyl and carboxyl groups) of GO facilitate the facile introduction of desired functional groups^22–27^, which enable adsorbent applications for removal of harmful elements. Due to these properties, many GO composites have been studied for the adsorption of heavy metals (Table 1). Herein, when focusing on the GO state prior to the functionalization process, there were various GO conditions, including solution, freeze-drying, oven-drying, etc. and some studies had no mention of it.

**Table 1:** Graphene oxide (GO) composite adsorbents synthesized using different GO drying processes.

Adsorbents	GO drying process	Contaminants	Ref.
Aminosilane-GO	No drying - solution	Cr(VI)	^28^
DCTA/E/GO	No drying	Cr(VI)	^35^
PAS-GO	Freeze-drying	Pd(II)	^36^
PAM-g-graphene	Freeze-drying	Pb(II)	^37^
TOA-EGO	Vacuum-oven-drying at room temperature	Cr(VI)	^38^
SAGO	Vacuum-oven-drying at room temperature	Cu(II), Pb(II)	^39^
AMGO	Oven-drying at 30 °C	Cr(VI)	^15^
PPy/GO	Vacuum-oven-drying at 40 °C	Cr(VI)	^40^
IT-PRGO	Vacuum-oven-drying at 60 °C	Hg(II)	^41^
PPy-GO	Oven-drying at 60 °C	Cr(VI)	^42^
Aminosilane-GO	Oven-drying at 100 °C	Cr(VI)	^43^
Chitosan/GO	Drying under unspecified condition	Cu(II), Pb(II), Cr(VI)	^24^
TGOCS	Not mentioned	Cr(VI)	^44^
PEI-GO	Not mentioned	Cr(VI)	^25^
GO-αCD	Not mentioned	Cr(VI)	^45^
MCGN	Not mentioned	Cr(VI)	^46^

Recently, we showed that the adsorbents synthesized by chemically bonding GO and amino-silanes have excellent performance in chromium adsorption (260.74 mg g^−1^)^25^. However, in the similar work by Janik *et al*. (2018), adsorbents have relatively low Cr(VI) adsorption capacities (13.3–15.1 mg g^−1^) and the opposite order of Lee *et al*. (2020)’s^28^. The minor yet remarkable difference between these two studies, which many researchers overlooked, is the use of different approaches for the preparation of GO. While Janik *et al*. (2018) used the oven-drying of GO at 100 °C (oven-dried GO (ODGO)) for preparation of GO, Lee *et al*. (2020) employed the solution form of GO (Solution-GO, SGO), which revealed the importance of adopting the appropriate drying process. As far as we know, there is no study suggesting suitable process of GO for surface functionalization so far.

Therefore, with an aim to find improved and effective processes for GO surface functionalization, we compared the differences in the Cr(VI) adsorption capacities caused by the variations in the conditions employed for the preparation of GO surfaces before the functionalization. We are certain that the results of this study will help researchers working with GO for the development of various applications.

## Results and discussion

### Cr(VI) adsorption isotherm on GO + 3N adsorbents

The recorded adsorption isotherms describe the physicochemical adsorption resulting from the interaction between Cr(VI) and the adsorbent surfaces. The obtained equilibrium data was fitted to the Langmuir and Freundlich isotherm models. The Langmuir equilibrium adsorption isotherm describes the adsorption reaction of the monolayer on homogenous surfaces as a function of the partial pressure at constant temperature. The equation for the Langmuir adsorption isotherm is shown in Eq. 1.

1 $${{Q}}_{{e}}=\frac{{{K}}_{{L}}{{Q}}_{{m}}{{C}}_{{e}}}{1+{{K}}_{{L}}{{C}}_{{e}}}$$

where Q_e_ is the equilibrium adsorption capacity (mg g^−1^); Q_m_ is the maximum adsorption capacity on the adsorbent surface of the monolayer (mg g^−1^); C_e_ is the equilibrium concentration of Cr(VI) (mg L^−1^); and K_L_ is the affinity constant (L mg^−1^)^29–31^.

Freundlich equilibrium adsorption isotherm describes the adsorption characteristics of the heterogeneous surface, which comprises the terminal and functional groups that offer stable binding sites. The Freundlich adsorption isotherm is an empirical equation and is represented by Eq. 2.

2 $${{Q}}_{{e}}={{K}}_{{F}}{{C}}_{{e}}^{1/{n}}$$

where Q_e_ is the equilibrium adsorption capacity (mg g^−1^); K_F_ is the Freundlich constant related to adsorption capacity (mg g^−1^); n is the adsorption intensity.

**Figure 1:**
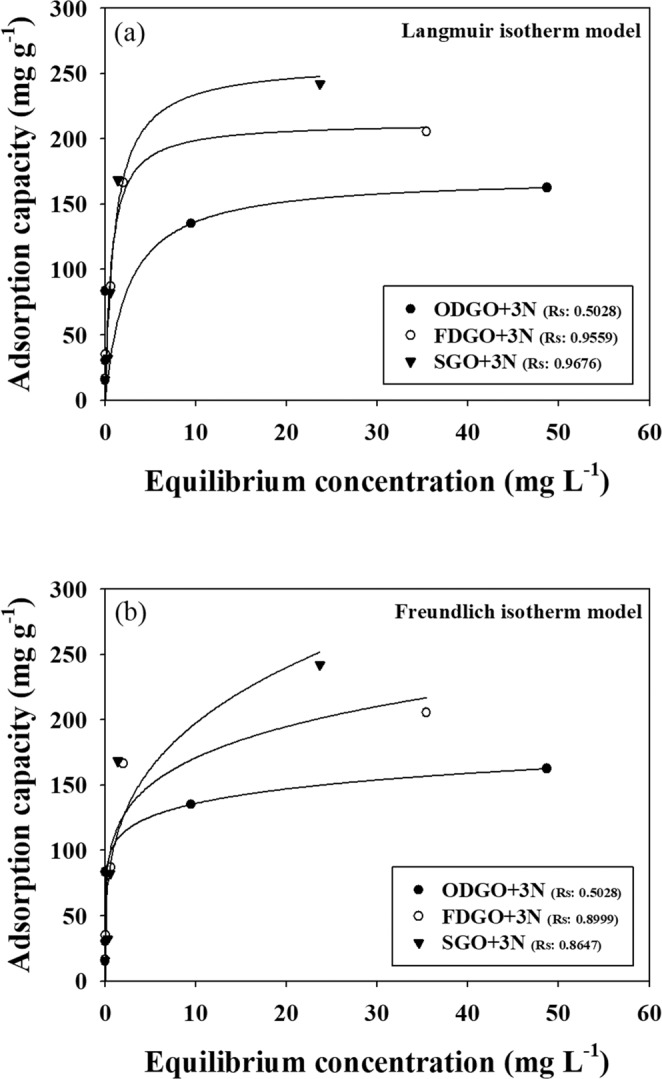
Cr(VI) adsorption isotherms for GO + 3N adsorbents: (**a**) Langmuir isotherm model and (**b**) Freundlich isotherm model.

The experimental results were fitted using Eqs. 1 and 2 described above. As shown in Fig. 1, the points and nonlinear curves were well matched. The last point in the Langmuir fitting (Fig. 1(a)) indicated that it was nearly in equilibrium. A comparison of the two models revealed that the Langmuir model showed relatively high regression coefficients (R square, Rs), whereas the Freundlich model showed low Rs values because of the high concentration results, indicating that the Langmuir model was relatively better in describing the adsorption capacities. These results confirmed that the surfaces of the adsorbents were homogeneous, and adsorption reactions occurred well in the monolayer. On the other hand, the isotherm models for ODGO + 3N did not fit the equilibrium adsorption data well, due to the poor fit of the third point of the result.

### Characterization

**Figure 2:**
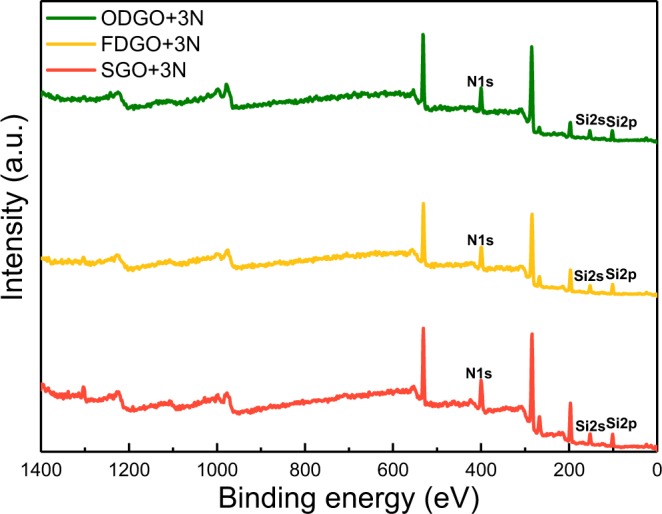
XPS survey scans of ODGO + 3N, FDGO + 3N and SGO + 3N.

Figure 2 shows XPS analysis after amine functionalization of the samples. It proved that amino silanes were successfully functionalized on the GO surface regardless of dry process. In adsorbents, the binding energies of Si atom were detected at around 153 eV (Si 2s) and 103 eV (Si 2p). Also, the peak of N 1 s at around 400 eV was confirmed^32^. Table 2 shows a comparison of the Cr(VI) maximum adsorption capacities of the adsorbents that were prepared by the different functionalization processes and showed a significant difference in adsorption capacities. SGO prepared without the drying process recorded the highest Cr(VI) adsorption capacity of 258.48 mg g^−1^, while ODGO dried at 70 °C in air showed the worst capacity of 173.45 mg g^−1^. The Cr(VI) adsorption capacity of FDGO was 212.46 mg g^−1^ and was in-between the values for the other two samples. The values for the 1/n data also showed the orders of adsorption for SGO, FDGO and ODGO. The larger the 1/n values, the greater is the adsorption. The data for the maximum adsorption capacities and 1/n values were therefore in agreement and indicate that the process is critical for imparting adsorption capacity to GO. During the functionalization process, the molecules used, access, react, and bind to the oxygen on the GO surface. The differences in the performances of the GO samples are expected to originate from the differences in the accessibility of the functionalized molecules to the Cr(VI) species; i.e., the overlapping of GO layers could contribute to the differences in the accessibility of the molecules to GO surface. To verify this hypothesis, the following additional analyses were carried out.

**Table 2:** Cr(VI) adsorption capacities of GO + 3N adsorbents.

	SGO + 3N	FDGO + 3N	ODGO + 3N
Q_m_ (mg g^−1^)	258.48	212.46	173.45
1/n	0.2807	0.1874	0.1120

**Figure 3:**
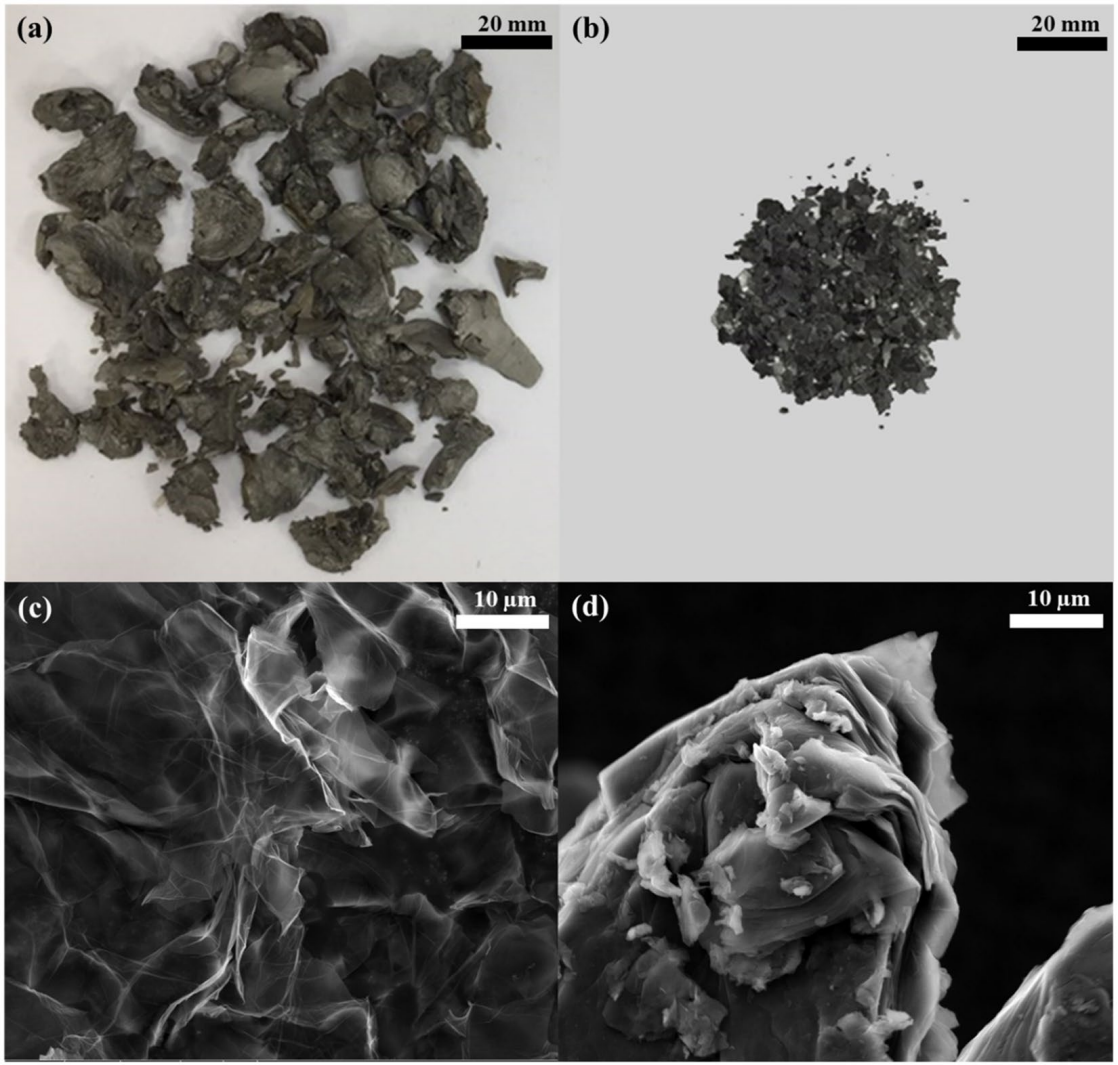
Optical images of (**a**) FDGO and (**b**) ODGO (200 mg) and SEM micrographs of (**c**) FDGO and (**d**) ODGO.

Figure 3(a,b) show the same amount of GO samples, which were prepared with different processes, and indicate a huge volume difference. FDGO had a much larger volume than ODGO. Further, SEM observations confirmed that the overlapping of GO layers is much more extensive in ODGO than FDGO (Fig. 3(c,d)). The layers of FDGO were well dispersed and enabled the visualization of the transparent GO layers and their wrinkles, whereas the layers of ODGO were multiply stacked and formed particles, which might have resulted in the exposed surface being smaller. The BET analysis results supported this observation. The surface area of ODGO was 2.9587 m^2^ g^−1^, which is significantly lower than the 24.139 m^2^ g^−1^ of FDGO (Table S1 in Supporting Information). In summary, oven-drying resulted in low dispersion of the GO layer, thereby reducing the accessible surface area. Next, we turned our attention to confirm that the reduction in the accessible surface area influenced the degree of functionalization, which is directly related to the adsorption performance.

To quantify the degree of functionalization, we conducted a TG analysis. TGA residual after heating up to 800 °C is the amount of SiO_2_, indicates that of functionalizing amino-silane on each GO adsorbent (Table 3). The residue obtained for SGO + 3N was the largest and was followed by FDGO + 3N and ODGO + 3N (Figure S1). Furthermore, the XPS analysis results were consistent with the TG results. The XPS atomic concentration results of Si/C and N/C indicated that the SGO + 3N contained the highest concentration of the amine functional groups on the surface. The TGA and XPS results have fewer differences than those of the BET surface area because the overlapped GO layers of ODGO might be dispersed further to some extent.

**Table 3:** Atomic concentration results from TGA and XPS.

	TGA residual (%)	N/C	Si/C
SGO + 3N	20.42	0.205	0.089
FDGO + 3N	16.24	0.179	0.074
ODGO + 3N	12.66	0.155	0.072

Elemental analysis was conducted to determine if the reduction of the exposed surface area was the only cause of performance degradation. The EA results presented in Table 4 confirm that the oxygen content was reduced due to partial reduction of the exposed surface area during the drying process, and was significantly lower in ODGO. This means that -COOH and -OH functional groups, which are necessary for functionalization, were reduced during the drying process, thus decreasing the density of silane-functionalization. These studies reveal that despite using the same GO source, variations in the drying processes cause the differences in the density of sites for silane-functionalization, as well as influence the effective surface area. In summary, the degradation of adsorption performance can be attributed to the physical changes, loss of effective GO surface area, the ensuing chemical changes, and the reduction of GO.

**Table 4:** Elemental analysis.

	GO dried at 25 °C	FDGO	ODGO
O/C ratio	0.74	0.68	0.59

## Conclusions

The studies revealed that avoiding drying process is the most effective approach for ensuring suitable surface functionalization of GO. This is not only because the dispersion of GO layers can be maintained and the effective exposure surface area can be extensive by avoiding the drying process, but the reduction of GO can be suppressed. More importantly, the drying method makes a significant difference in the GO surface state. If the drying process is inevitable, freeze-drying is recommended rather than oven-drying. General oven-drying has a negative effect on the GO’s performance, caused by the overlapping of GO layers and the reduction of GO. It is confirmed that freeze-drying causes less overlapping and lower loss of oxygen-containing surface functional groups, which act as bonding-sites for silane-functionalization. In this paper, we suggest suitable processes for the treatment of GO with systematic experiments and analysis, which will aid better functionalization and development of GO-based catalysts.

## Materials and methods

### Materials

Graphene oxide (GO)-V50 was purchased from Standard Graphene. 3-[2-(2-aminoethylamino)ethylamino]propyl-trimethoxysilane (AEAEAPTMS, technical grade), methanol (≥99.9%), and Cr(VI) stock solution dissolving potassium chromate (K_2_Cr_2_O_7_, 99.5%) in 0.01 M nitric acid (HNO_3_) were obtained from Sigma-Aldrich and Kanto chemical.

### Synthesis of 3N-GO-adsorbents

**Scheme 1:**
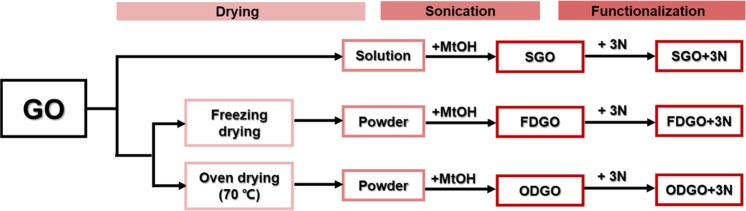
Flowchart of the synthesis of GO + 3N adsorbents.

Scheme 1 shows the overall processes used for the adsorbent synthesis and summarizes the differences by the process. GO-V50 was functionalized with AEAEAPTMS using a slight modification of a reported method^10,33,34^, and 3 types of GO-V50 were prepared. The first was prepared in the solution form, the second was prepared by freeze-drying, and the final was by oven-drying at 70 °C Each of these suspensions (0.5 g of GO/140 mL of methanol) was sonicated for 2 h and refluxed at 60 °C with stirring at 1000 rpm. At 60 °C, AEAEAPTMS (5 mL) was added to each suspension. After a 24 h reaction, the product was washed with ethanol using centrifugation at 13,500 rpm for 12 min three times. Each washed sample of AEAEAPTMS-GO (3N-GO) was dried at 40 °C overnight. Lastly, the products were treated with 0.1 M HCl for 6 h and were collected by centrifugation and dried at 40 °C overnight.

### Equilibrium adsorption of Cr(VI) on GO-adsorbents

We acquired the Cr(VI) adsorption isotherms of five samples of Cr(VI) solutions of concentrations 5, 10, 25, 50, 100 mg∙L^−1^. The three samples (15 mg each) of the adsorbents which were prepared by drying at 70 °C, freeze-drying, and as a GO solution, respectively, and were injected into each of the five Cr(VI) solutions and were reacted for 24 h at 25 rpm using a rotator. Upon reaction completion, the solution was centrifuged at 4200 rpm for solid-liquid separation, and the mixture was filtered using a 0.2 μm PVDF syringe filter for completely separating the solution from the adsorbents.

### Scanning electron microscopy (SEM)

The morphology of GO samples was analyzed by scanning electron microscopy (SEM, FEI Inspect F50, AP-tech Company).

### Brunauer-Emmett-Teller (BET)

The specific surface area was determined from the linear portion of BET plots (P/P_0_ = 0 to 1), which were acquired using a surface area analyzer (BEL-SORP-max, BEL Japan Inc., Japan).

### Thermogravimetric analysis (TGA)

Thermogravimetric analysis (TGA, N-1500, Scinco) of the GO-adsorbents was performed to confirm the amount of aminosilanes grafted onto the GO surface by heating the adsorbents from 100 to 800 °C at a rate of 10 °C min^−1^ under air condition.

### X-ray photoelectron spectroscopy (XPS)

X-ray photoelectron spectroscopy (XPS, PHI 5000 VersaProbe Ulvac-PHI with Al X-ray monochromatic source (Al K_α_ source with energy 1486.6 eV at 24.5 W), Physical Electronics Inc.) analysis was performed to determine the surface composition of the adsorbents. The binding energies were referenced to the C 1s line at 284.6 eV from the adventitious carbon.

### Elemental analysis (EA)

The elemental ratio (O/C ratio) of GO was obtained from elemental analysis (EA, FLASH 2000, Thermo Scientific). The average values of 2~3 measurements were used as the data.

### Inductively coupled plasma optical emission spectrometer (ICP-OES)

An inductively coupled plasma optical emission spectrometer (ICP-OES, ProdigyPlus, Prodigy) and an autosampler were used for the quantitative analysis of the Cr(VI) solution before and after the reaction. Each sample was analyzed with 4 replicates, and the results were interpreted as the average value.

## Data Availability

All data generated and/or analyzed during this study are included in this published article.

## Supplementary informataion

Supplementary informataion.
