# Supplementary material for: Performance Differences of Hexavalent Chromium Adsorbents Caused by Graphene Oxide Drying Process
Source: Sci Rep. 2020 Mar 17;10:4882. doi: 10.1038/s41598-020-61760-2 (PMC7078186; doi:10.1038/s41598-020-61760-2)
Supplement: Supplementary file 1 — Supplementary informataion. [file 41598_2020_61760_MOESM1_ESM.docx]

***Supplementary Materials***

Performance Differences of Hexavalent Chromium Adsorbents Caused by Graphene Oxide Drying Process

JinHyeong Lee^1,2†^, Hee-Gon Kim^2,3†^, Jung-Hyun Lee^2^, So-Hye Cho^1,4^, Kyung-Won Jung^3^, Seung Yong Lee^1,4*^ & Jae-Woo Choi.^3,5*^

^1^ Materials Architecturing Research Center, Korea Institute of Science and Technology, Hwarang-ro 14-gil 5, Seongbuk-gu, Seoul 02792, Republic of Korea

^2^ Department of Chemical and Biological Engineering, Korea University, 145 Anam-ro, Seongbuk-gu, Seoul 02841, Republic of Korea

^3^ Water Cycle Research Center, Korea Institute of Science and Technology, Hwarang-ro 14-gil 5, Seongbuk-gu, Seoul 02792, Republic of Korea

^4^ Division of Nano & Information Technology, KIST school, Korea University of Science and Technology, Hwarang-ro 14-gil 5, Seongbuk-gu, Seoul 02792, Republic of Korea

^5^ Division of Energy & Environment Technology, KIST school, Korea University of Science and Technology, Hwarang-ro 14-gil 5, Seongbuk-gu, Seoul 02792, Republic of Korea

* Corresponding authors, Seung Yong Lee, e-mail*:* [patra@kist.re.kr](mailto:patra@kist.re.kr) (S.-Y. Lee); Jae-Woo Choi, [plead36@kist.re.kr](mailto:plead36@kist.re.kr) (J.-W. Choi).

† These authors contributed equally to this work.

Table S1

Physical characteristics of FDGO and ODGO.

|  | **a_s,BET_**  **[m^2^ g^-1^]** | **Total pore volume [cm^3^ g^-1^]** | **Mesopore volume [cm^3^ g^-1^]** | **Micropore volume [cm^3^ g^-1^]** | **Micropore size distribution [nm]** |
| --- | --- | --- | --- | --- | --- |
| FDGO | 24.139 | 0.1346 | 0.1324 | 0.0009 | 1.9 |
| ODGO | 2.9587 | 0.0159 | 0.0155 | -0.0002 | 1.9 |

******

Figure S1. TG analysis curves of ODGO+3N, FDGO+3N and SGO+3N.
